# Attachment to God and meaning in life: the role of synchronicity awareness among ultra-Orthodox and secular individuals

**DOI:** 10.3389/fpsyg.2026.1609435

**Published:** 2026-03-11

**Authors:** Pninit Russo-Netzer, Tamar Icekson

**Affiliations:** 1 Community, Meaning and Healing (CMH) Research and Development Center, Faculty of Psychology, Achva Academic College, Arugot, Israel; 2 School of Education, Department of Management, Ben-Gurion University of the Negev, Be'er Sheva, Israel; 3 Program in Organizational Development & Consulting, School of Behavioral Sciences, Peres Academic Center, Rehovot, Israel

**Keywords:** attachment to God, meaning in life, secular Jews, synchronicity awareness, ultra-Orthodox

## Abstract

**Introduction:**

This study aimed to explore cultural variations in meaning in life (MIL) by comparing ultra-Orthodox and secular individuals in Israel, examining the roles of attachment to God and synchronicity awareness.

**Method:**

A matched sample of ultra-Orthodox and secular participants completed measures of attachment to God, synchronicity awareness, synchronicity meaning-detection, and MIL. Participants were matched on age and gender using nearest neighbor matching. Measures included the Experiences in Close Relationships scale adapted for God attachment, the Synchronicity Awareness and Meaning-Detecting Scale, and the Meaning in Life Questionnaire. Path analysis was used to test the hypothesized relationships.

**Results:**

Ultra-Orthodox individuals reported higher levels of anxious attachment to God and MIL, while secular individuals exhibited more avoidant attachment. Both groups showed similar levels of synchronicity awareness and meaning-detection. Insecure attachment styles to God (anxious and avoidant) were negatively associated with MIL. Mediation analyses indicated that synchronicity meaning-detection significantly mediated the relationship between anxious attachment to God and MIL, suggesting that interpreting coincidences as meaningful may enhance overall life meaning for those with anxious attachment. Synchronicity awareness did not mediate this relationship, highlighting the importance of active interpretation rather than mere recognition of events.

**Discussion:**

The findings highlight the complex interplay between religious affiliation, attachment to God, and synchronicity experiences in shaping perceptions of life meaning across different cultural contexts. The study extends attachment theory and synchronicity research, offering insights into meaning-making processes and wellbeing. Future research should employ longitudinal and experimental designs to further validate these relationships and explore implications for enhancing MIL across diverse populations.

## 1 Introduction

Meaning in life (MIL) is a crucial factor in living a fulfilling life. The current integrative conceptualization of MIL encompasses three primary dimensions: comprehension, purpose, and mattering (George and Park, 2016; Martela and Steger, 2016). Specifically, MIL can be described as the extent to which individuals perceive their lives as coherent, guided by meaningful goals, and having significance in the broader world—each of these reflecting the three core dimensions (George and Park, 2016, p. 2). Grounded in this conceptual framework, a growing body of empirical research over the past few decades has reinforced the theoretical understanding that MIL is central to human experience. It is increasingly recognized as a key contributor to human flourishing and as a coping mechanism for adapting to life's challenges and suffering (e.g., Czekierda et al., 2017; Russo-Netzer and Vos, 2026). However, despite this expanding research and deeper understanding of MIL—its sources and its influence on human functioning—much of the focus remains on the individual level. Yet the experience and manifestation of meaning can vary significantly across different contexts, with various sociocultural factors likely affecting the sources and processes of MIL (Steger et al., 2008). Religion can be seen as such a sociocultural framework that facilitates meaning-making processes for its members (Dahinden and Zittoun, 2013; Park, 2005).

In institutionalized religions, overarching narratives are often sustained and reinforced through ideologies, practices, coping mechanisms, symbols, community support, and shared beliefs and traditions (e.g., Pargament and Mahoney, 2009). Such a framework contributes to an individual's sense of control, purpose in life, and overall security, ultimately fostering positive psychological outcomes and wellbeing (Park, 2005; Silberman, 2005). Religions further provide an organized worldview, instilling a coherent set of values, standards, and guidelines for living a meaningful and worthwhile life (Krok, 2014). Through shared belief systems and worldviews that offer moral guidance from birth to death and beyond, religions also provide clear directives on beliefs and values. However, less is known about the psychological mechanisms that may be in play in the relationship between such cultural-religious structures and an individual's sense of meaning in his or her life (Abu-Raiya et al., 2020; Steger and Frazier, 2005).

Moreover, given that much of the research on meaning in life has been conducted in North America and Europe, calls have emerged to extend knowledge beyond specific cultural backgrounds (what has been termed as WEIRD: Western, educated, industrialized, rich, and democratic; Diener et al., 2018; Henrich et al., 2010) and include more international and diverse samples (Russo-Netzer and Vos, 2026). Thus, the present study aims to expand the scope of understanding cultural differences in levels of meaning in life, by comparing ultra-Orthodox religious individuals to their secular counterparts, and to examine two underexplored factors that may be in play in such experiences: one's sense of connection with the transcendent (i.e., attachment to God) and synchronicity awareness.

### 1.1 Meaning in life among Jewish ultra-orthodox individuals

Compared to other religions such as Christianity, Judaism places a greater emphasis on actions rather than thoughts, prioritizing practice over beliefs and religious participation over intrinsic motivation (e.g., Cohen and Hill, 2007; Pirutinsky, 2009). Beyond such differences, substantial variations also exist between Orthodox and non-Orthodox Jews (Cohen et al., 2003).

The diverse population of Israeli society is a fertile field for cross-cultural research, particularly in comparing Orthodox and non-Orthodox Jews. In Israel, ultra-Orthodox society adheres to Jewish tradition and *halakha* (religious law) and separates itself from lifestyles or worldviews that contradict its values (Brown, 2017). It is predicted that by 2030, 16% of the population in Israel will be ultra-Orthodox Jews, compared to about 10.6% to 12.3% of the population being ultra-Orthodox today. Secular Jews make up 44.4% of the national population, while the rest of the Jewish population identify themselves as religious (Central Bureau of Statistics, 2018; Malach and Cahaner, 2020). Compared to the secular Jewish population, the ultra-Orthodox Jewish population in Israel has lower socioeconomic status, lower monthly income, and higher poverty. Due to their community values, ultra-Orthodox men have low formal secular education and high unemployment rates (Malach and Cahaner, 2020). Compared to secular women, ultra-Orthodox women are more likely to work part-time, low-paying jobs. Families with ultra-Orthodox members have 6.6 children on average, compared with 2.1 among secular Jewish families (Central Bureau of Statistics, 2018; Malach and Cahaner, 2020). Nevertheless, the ultra-Orthodox community is consistently reported as having higher levels of life satisfaction and lower depression and anxiety rates than other religious and secular Jewish groups in Israel (Russo-Netzer et al., 2021; Schellekens, 2019).

Previous research conducted on the ultra-Orthodox in Israel suggested their high life satisfaction was linked with social community, as well as behaviors that contributed to a sense of meaning in life (Klonover et al., 2023; Russo-Netzer and Bergman, 2020) and gratitude (Russo-Netzer et al., 2021). Building on such previous endeavors, the present study suggests that ultra-Orthodox individuals will report higher levels of MIL compared with their secular counterparts.

### 1.2 Attachment to God and meaning in life

Bowlby's (1982/1969) attachment theory suggests that children develop emotional bonds with important older individuals, known as attachment figures, who ideally offer a secure and caring environment (e.g., Ainsworth et al., 2015). When these figures are consistently present and responsive, children feel safe, fostering a secure attachment style. Conversely, if attachment figures are neglectful or unresponsive, children may begin to doubt their own worthiness of love and the reliability of others. Maladaptive interpersonal patterns formed in early childhood due to abusive or neglectful attachment figures often lead to the development of insecure attachment styles (Bowlby, 1982/1969). These attachment styles function as mental schemas or working models, shaping expectations for social interactions and functioning, including emotion regulation, interpretation of others' actions, and behavioral responses (e.g., Mikulincer and Shaver, 2019).

Two primary insecure attachment styles are anxious and avoidant. Anxious attachment involves heightened efforts to maintain closeness, dependency, constant monitoring of relationships, and clinginess. Avoidant attachment is marked by avoiding self-disclosure and emotional intimacy, independence from others, and a tendency to shy away from confronting relational conflicts (Brennan et al., 1998; Mikulincer and Shaver, 2019).

Previous work has suggested that attachment plays a crucial factor in the development and sustenance of meaning throughout life (e.g., Bodner et al., 2014; Dewitte et al., 2019; Lopez et al., 2015; Mikulincer and Shaver, 2013). Early attachment experiences lay the groundwork for recognizing patterns and fostering a sense of order and coherence. Interactions with responsive attachment figures further enhance mentalization abilities and promote exploration of both the inner and outer worlds, allowing individuals to form complex representations of themselves, others, and their surroundings, which in turn shapes how they interpret meaning in their lives (Dewitte et al., 2019).

Additionally, attachment security acts as a lasting and powerful resource in managing threats to meaning. When faced with disruptions, secure attachment offers a consistent set of representations, helping individuals maintain or restore their sense of order and purpose during difficult times. Secure attachment also fosters cognitive openness and tolerance for ambiguity, allowing for flexible and realistic adjustments to meaning when confronted with conflicting information (Dewitte et al., 2019).

Taking it a step further, the model of religion-as-an-attachment process suggests that a relationship with God can be described as a significant attachment bond (Kirkpatrick and Shaver, 1992; Kirkpatrick, 2005). Thus, a connection to the transcendent may function as a “secure base” from which to explore and a “safe haven” that comforts and reassures individuals (e.g., Cherniak et al., 2021; Granqvist et al., 2012). Internal working models resulting from interpersonal experiences generalize to representations of God. More specifically, the model points out the resemblance between internal working models of God and religion and those developed within close relationships, suggesting that securely attached individuals perceive positive God images as “reliable and trustworthy” (Kirkpatrick, 1998; p. 962). The unmediated connection with the transcendent, experienced as a subjective sense of personal providence, connection, and guidance, especially among the ultra-Orthodox, reflects such a connection.

Indeed, it has been found that insecure attachment to God is positively related to psychological distress (e.g., Stulp et al., 2019; Tung et al., 2018). These patterns were documented in cross-sectional studies conducted among Christian, Muslim, and Jewish samples (Hiebler-Ragger et al., 2016; Miner et al., 2017; Pirutinsky et al., 2019), as well as in longitudinal ones (Calvert, 2010; Wilt et al., 2019). Moreover, a few studies have pointed to the positive association between secure attachment to God and a sense of purpose and meaning in life (e.g., Culver and Lundquist Denton, 2017; Keefer and Brown, 2018; Stroope et al., 2013). Taken together, we hypothesized that insecure attachment styles to God (avoidant and anxious) would be directly and negatively associated with meaning.

### 1.3 Synchronicity awareness as a possible mediator between attachment to God and meaning in life

A key aspect of finding MIL is the experience of coherence—a feeling that life “makes sense” and forms a cohesive whole (George and Park, 2016; Heintzelman and King, 2014; Martela and Steger, 2016). People are driven to perceive structure in their surroundings (e.g., Frankl, 1963) and tend to prefer clarity and order over ambiguity and uncertainty (e.g., Heine et al., 2006). Jung's (1969) concept of synchronicity—instances of meaningful coincidence that appear to be beyond mere chance—may illustrate this human tendency.

Synchronicity is broadly defined as the connection of the inner and outer worlds through unusual and meaningful coincidences. For example, one might be preoccupied with a thought and later encounter an event that remarkably aligns with that inner state. Jung (1969) described synchronicity as unpredictable occurrences of meaningful coincidence, reflecting “the coincidence of events in space and time as meaning something more than mere chance” (Jung, 1950/1997, p. 25). Since the introduction of the concept, interest in synchronicity has grown significantly (Hocoy, 2012; Sacco, 2019). Clinical case studies suggest that recognizing synchronicity can be beneficial in therapeutic contexts (e.g., Roxburgh et al., 2018) and in understanding career paths and decisions (e.g., Guindon and Hanna, 2002).

A recent integrative heuristic model, the REM model, characterizing such experiences has been suggested (Russo-Netzer and Icekson, 2020). This model consists of three core components: receptiveness (R), which entails heightened awareness and openness to both internal and external experiences, laying the groundwork for an exceptional encounter (E). An exceptional encounter refers to an unforeseen event that resonates with an individual's inner state, triggering distinct and memorable emotions. Lastly, meaning-detection (M) involves the deliberate process of connecting the event to one's personal life narrative, through which individuals derive meaning from their synchronicity experiences by uncovering a hidden order—an organizing framework that makes life more comprehensible and reinforces the belief that events carry deeper significance. Based on this model, a validated tool was developed to assess individual differences in experiencing synchronicity, focusing on two key aspects: awareness and meaning-detection (Russo-Netzer and Icekson, 2023).

Using the Synchronicity Awareness and Meaning-Detection Scale, recent studies pointed to some significant associations between the experience of meaningful coincidences and human brain functioning (Rominger et al., 2023, 2024c), as well as to openness to experience, creativity, mindfulness, and meditation practice (Butzer et al., 2024; Rominger et al., 2024a; Rosenstreich et al., 2024; Russo-Netzer and Icekson, 2023). While awareness of synchronicity experiences is rather widespread (e.g., Fach et al., 2013; Russo-Netzer and Icekson, 2023), less examined is the possibility that content and interpretation of synchronic experiences may be subject to the sociocultural background of individuals. For example, findings from a qualitative study have suggested that religious participants attributed their experiences to the transcendent (Russo-Netzer and Icekson, 2020). This sociocultural variation calls for further unpacking.

Building on the religion-as-an-attachment theory, insecure attachment to the transcendent may destabilize one's sense of order and coherence (Kirkpatrick and Shaver, 1992; Kirkpatrick, 2005). As such, insecure attachment often involves a heightened sensitivity to feelings of uncertainty. Insecure attachment may also involve feelings of mistrust or disconnectedness (Mikulincer and Shaver, 2019). In this context, awareness of and detecting meaning in synchronicity experiences may serve as a compensatory mechanism. Observing and interpreting coincidences as meaningful, when a sense of connection to the transcendent is fragile or unstable, may provide a reassuring sense of guidance and purpose. Furthermore, paying attention to synchronicities can also provide a sense of validation that things are unfolding as they should, offering comfort in the face of doubt. Provisionally, positive correlations were reported between synchronicity awareness (SA) and meaning-detection (MD), optimism, and positive affect (Rominger et al., 2024b; Russo-Netzer and Icekson, 2020, 2023). Moreover, SA and MD was suggested as a pathway to enhance a sense of MIL (Russo-Netzer and Icekson, 2020).

### 1.4 The current study

**Figure 1:**
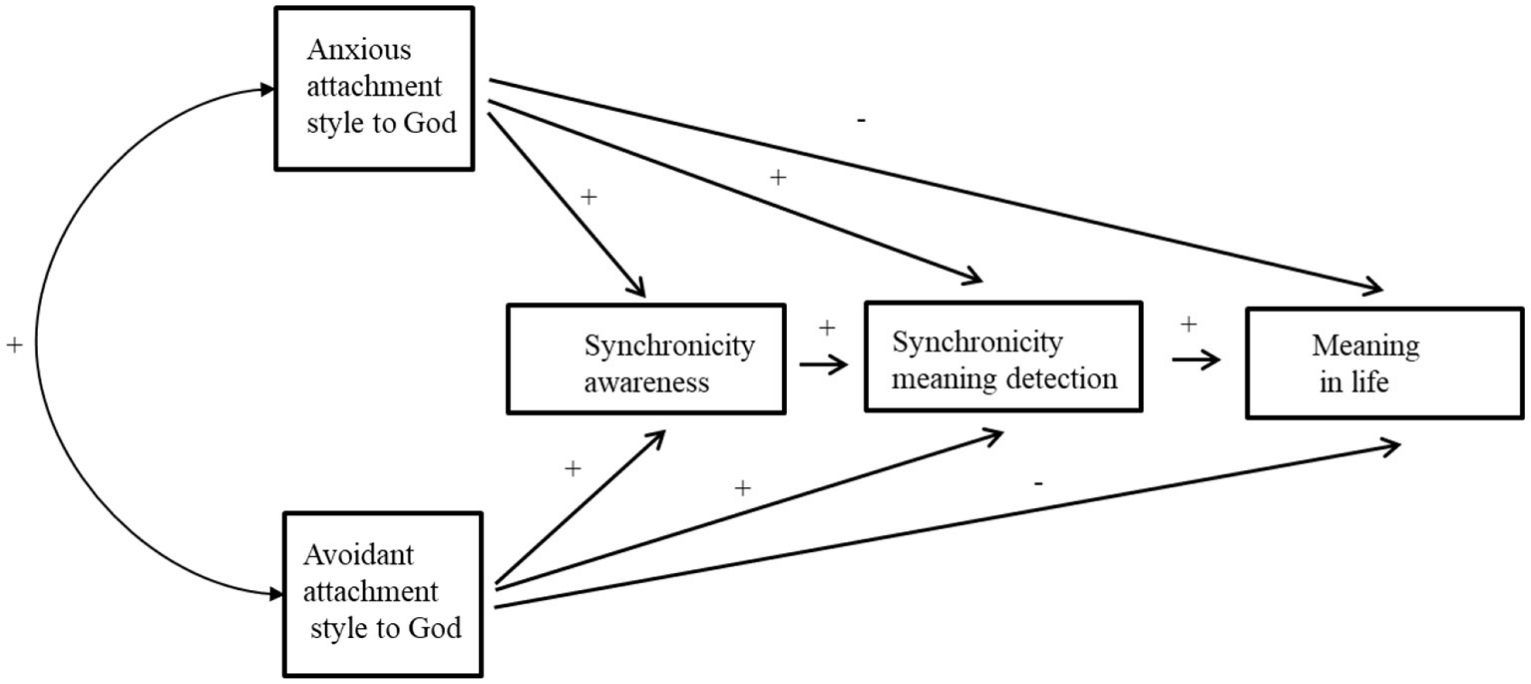
The theoretical model.

This study aimed to explore the psychological mechanism underlying the relationship between attachment to God and sense of MIL as mediated by SA and MD. Given the limited knowledge about the similarities and differences between ultra-Orthodox and secular individuals in relation to attachment to God, SA, and MIL, the first goal was to explore these associations. The second goal was to deepen our understanding of the relationships between attachment to God, SA, and MIL. Our hypotheses were (see Figure 1):

**H1**: Anxious attachment to God will be directly and negatively associated with presence of MIL.

**H2**: Avoidant attachment to God will be directly and negatively associated with the presence of MIL.

**H3**: Anxious attachment to God and the presence of MIL will be mediated by higher levels of SA and subsequent higher levels of synchronicity MD, leading to higher MIL.

**H4**: Avoidant attachment to God and the presence of MIL will be mediated by higher levels of SA and subsequent higher levels of synchronicity MD, leading to higher MIL.

**H5**: Anxious attachment style to God and the presence of MIL will be mediated by higher levels of synchronicity MD, leading to higher MIL.

**H6**: Avoidant attachment style to God and the presence of MIL will be mediated by higher levels of synchronicity MD, leading to higher MIL.

## 2 Method

### 2.1 Transparency and openness

To estimate a priori-required sample size, Monte Carlo software with the following statistical assumptions was used: type 1 error of 5% and minimum statistical power of 80% (Lakens, 2022; Perugini et al., 2018). Based on previous studies, moderate associations were expected between the study variables (Russo-Netzer et al., 2021; Russo-Netzer and Icekson, 2023). For this reason, the minimum sample size was estimated at 235 respondents. All data, analysis code, and research materials are available upon request from the authors. Statistical analyses were conducted using the SPSS program version 28 and AMOS program version 28 (Arbuckle, 2019; IBM Corp, 2021). This study's design and its analysis were not pre-registered.

### 2.2 Participants

The final cohorts for analysis included 121 ultra-Orthodox Jewish Israeli participants and 121 matched secular Jewish Israeli participants. The matching was performed exclusively on age and gender. Table 1 presents the comparison between the groups before and after the matching procedure. As shown there, after the matching, groups were similar in age and gender. Each sample included 46.3% males and 53.7% females, with a mean age of 29.54 years (SD = 10.75).

**Table 1:** Comparison between the groups before and after the matching procedure.

**Variable**	**Haredi-orthodox participants**		**Seculars participants**		***p***
	***N*** **(%)**	***M (SD)***	***N*** **(%)**	***M (SD)***	
**Before matching**					
Gender					0.837
• Males	79 (40.1%)		90 (41.1%)		
• Females	118 (59.9%)		129 (58.9%)		
Age		28.24 (10.64)		30.97 (10.44)	0.007
**After matching**					
Gender					1.000
• Males	56 (46.3%)		56 (46.3%)		
• Females	65 (53.7%)		65 (53.7%)		
Age		29.54 (10.75)		29.54 (10.75)	1.000

### 2.3 Procedure

Ultra-Orthodox participants were recruited by a graduate student research assistant with an ultra-Orthodox background, using a snowball sampling technique. The original sample included 217 ultra-Orthodox participants. In order to collect data from an equivalent secular sample, an Israeli paid survey platform acknowledged by the Israeli Bureau of Statistics as representing the Israeli population was used. The panel consists of more than 50,000 people over the age of 18 who signed up to participate in paid internet surveys. Recently, online panels have become a common and valid way to target and reach respondents in social science research (e.g., Zheng et al., 2023). Participants from the panel received compensation of $5 for filling out the questionnaires. The original secular sample included 219 participants.

All participants completed a series of online questionnaires after signing an informed consent form, which specified the purpose of the research, its procedures, and the voluntary nature of participation. Participants were guaranteed anonymity, and no disclosure of personal details was required. The study was approved by the IRB in the first author's academic institution (IRB number 2023121).

### 2.4 Instruments

The internal reliabilities of all scales used in the current study, assessed with both Cronbach's alpha and McDonald's omega, were good to excellent and are reported in Table 2.

**Table 2:** Internal reliabilities, means, standard deviations, and zero-order intercorrelations of variables (*N* = 242).

**Variable**	**α**	**ω**	***M***	***SD***	**1**	**2**	**3**	**4**
1. Synchronicity awareness	0.83	0.84	2.28	0.96	–			
2. Synchronicity meaning-detection	0.91	0.91	4.54	1.22	0.64^**^	–		
3. Presence of meaning in life	0.87	0.88	4.83	1.39	0.23^**^	0.28^**^	–	
4. Attachment to God anxiety	0.85	0.87	2.43	1.17	0.14^*^	0.24^**^	−0.02	–
5. Attachment to God avoidance	0.96	0.96	3.39	1.83	−0.14^*^	−0.21^**^	0.50^**^	−0.31^**^

M and SD are used to represent mean and standard deviation, respectively.

^*^p < 0.05; ^**^p < 0.01.

#### 2.4.1 Experiences in close relationships (ECR) scale

Attachment to God was measured using the Granqvist et al. (2012) adaptation of the Hebrew version of Brennan et al. (1998) ECR scale (Mikulincer and Florian, 2000). The original scale aimed to measure attachment avoidance and attachment anxiety in interpersonal relationships. The 20-item Granqvist et al. (2012) measure is used to assess avoidance and anxiety in regard to God as an attachment figure. A 10-item subscale measures each dimension and employs a 7-point Likert-type scale (1 = strongly disagree to 7 = strongly agree). Participants are asked to describe how they often feel when in close relationships with God (e.g., attachment avoidance subscale: “I feel comfortable being close to God”; attachment anxiety subscale: “I worry about being abandoned by God”).

#### 2.4.2 Synchronicity awareness and meaning-detecting (SAMD) scale

The SAMD scale is comprised of two subscales: SA and synchronicity MD (Russo-Netzer and Icekson, 2023). The 9-item SA subscale refers to awareness of the occurrence of synchronicity events in daily lives. (e.g., “I ran into something or someone that I thought about in an unexpected place”) and uses a 6-point scale (0 = never, 1 = once, 2 = twice or more, 3 = rarely, 4 = often, 5 = all the time). The 13-item MD subscale refers to the meaning detected in synchronicity events or experiences (e.g., “I believe that unexplained events enable new discovery and development”) and uses a 7-point Likert scale (1 = not at all to 7 = to a high degree).

#### 2.4.3 Meaning in life questionnaire (MLQ)

The validated Hebrew version (Littman-Ovadia and Steger, 2010) of the 5-item presence of meaning in the individual's life subscale was used to assess MIL (MLQ-P; e.g., “I understand my life's meaning” and “My life has no clear purpose”) (Steger et al., 2006). The items were rated using a 7-point Likert scale (1 = absolutely untrue to 7 = absolutely true).

In addition to these questionnaires, demographic information was also collected from all of the participants.

### 2.5 Data preparation

In order to control observed confounding variables, we used nearest neighbor matching (NNM) (Dang et al., 2021) to create an ultra-Orthodox comparison group that most closely resembled the secular group as to age and gender. We used the “case-control matching” procedure in SPSS, with match tolerance of 0.3 for both age and gender. After performing the matching procedure, we assessed the balance of covariates between the groups using standardized differences.

### 2.6 Data analysis

Normality of the study variables was examined using descriptive statistics and Kolmogorov–Smirnov tests (Demir, 2022). Descriptive statistics indicated that all variables demonstrated acceptable levels of skewness (|skew| < 1, ranging between −0.53 to 0.80) and kurtosis (|Kurtosis| < 2, ranging between −1.32 to 0.20), suggesting only mild deviations from normality. These thresholds are consistent with commonly used guidelines that treat skewness and kurtosis values within approximately −2 to +2 as indicative of acceptable univariate normality for many parametric analyses (Demir, 2022). Kolmogorov-Smirnov tests indicated statistically significant departures from normality only for attachment anxiety to God (D = 0.11, *p* = 0.005) and attachment avoidance to God (*D* = 0.12, *p* = 0.002). Synchronicity awareness, meaning detecting and meaning presence did not significantly deviate from normality (all *p-values* > 0.05). Given the moderate sample size (*n* = 241), the small magnitude of these distributional departures, and the analytic strategy, which relied primarily on bootstrap confidence intervals for estimating indirect effects, the analyses were judged to be reasonably robust to violations of normality assumptions.

To examine the differences and similarities between the two groups, we first conducted a series of independent-samples *t*-tests with Bonferroni's correction for multiple comparisons (see Table 3). Then we examined pairwise correlations among all study variables (see Table 2). Statistical analyses then were conducted using SPSS and AMOS programs, version 28 (Arbuckle, 2019; IBM Corp., 2021).

**Table 3:** Results of independent sample tests between the secular and the ultra-Orthodox samples.

**Variable**	***Ultra-Orthodox***		***Secular***		***t* (240)**	***p***	**Cohen's *d***
	***M***	***SD***	***M***	***SD***			
1. Synchronicity awareness	2.32	0.87	2.23	1.04	0.70	1.00	0.09
2. Synchronicity meaning-detection	4.54	1.16	4.55	1.29	−0.05	1.00	−0.01
3. Presence of meaning in life	5.37	1.35	4.30	1.22	6.45	< 0.001	0.83
4. Attachment to God anxiety	2.61	0.98	2.24	1.30	2.48	0.07	0.32
5. Attachment to God avoidance	2.14	1.20	4.64	1.47	−14.46	< 0.001	−1.86

All *p*-values were calculated using Bonferroni's correction for multiple comparisons.

Several goodness of fit indices were used to explore the fit of the model to the data (Hoyle and Panter, 1995; Mueller and Hancock, 2019). Three absolute fit indices were used: the χ^2^ statistic Root Mean Square Error of Approximation (RMSEA), and the SRMR. Three additional relative fit indices were used: the Normed Fit Index (NFI), the Comparative Fit Index (CFI), and the Tucker-Lewis Index (TLI). An expected cross validation index (ECVI) was also calculated for the model. A non-significant χ^2^ statistic, RMSEA and SRMR scores below 0.06, as well as NFI, CFI, TLI values above 0.95, indicate excellent fit, whereas SRMR values below 0.08 and NFI, CFI, TLI above 0.90 indicate adequate fit. Then ECVI were evaluated, since lower values are considered better.

Finally, to explore the research hypotheses we used a path analysis model (Hayes et al., 2017). As a part of the model test, we likewise tested for the significance of the indirect effects to determine if suggested mediation effects would take place.

## 3 Results

Surprisingly, as can be seen in Table 3, no significant differences were found between the SA scores of the ultra-Orthodox group (*M* = 2.31, *SD* = 0.88) and the secular group (*M* = 2.23, *SD* = 1.04). Moreover, no significant differences were found between the MD scores of the ultra-Orthodox group (*M* = 4.54, *SD* = 1.16) and the secular group (*M* = 4.55, *SD* = 1.29). However, significant differences were found between the two groups in all of the other variables. Presence of MIL in the ultra-Orthodox group was significantly higher (*M* = 5.37, *SD* = 1.35) than in the secular group (*M* = 4.30, SD = 1.22), with a large effect size (*d* = 0.83). Regrading attachment to God, ultra-Orthodox participants reported on higher levels of attachment anxiety (*M* = 2.61, SD = 0.98) than secular participants (*M* = 2.24, SD = 1.30), with a small to medium effect size (*d* = 0.32). Secular participants reported on higher levels of attachment avoidance (*M* = 4.64, *SD* = 1.47) than ultra-Orthodox participants (*M* = 2.14, *SD* = 1.20), with a large effect size (*d* = −1.86).

The prevalence of synchronicity experiences in both groups was high. In the secular group, all participants reported encountering at least one synchronicity experience. In the ultra-Orthodox group, 98% reported on at least one synchronicity experience (only two participants reported they had never experienced one).

Regarding descriptive statistics, Table 2 presents the internal reliabilities (Cronbach's alpha), means, and standard deviations of the variables. In addition, to estimate the associations between the study's variables, an analysis of all pairwise Pearson correlations was conducted. The analysis revealed that SA was significantly and positively correlated with synchronicity MD (*r* = 0.64, *p* < 0.001(, presence of MIL (*r* = 0.23, *p* < 0.001(, and anxious attachment to God (*r* = 0.14, *p* = 0.03). SA was significantly and negatively correlated with avoidant attachment to God (*r* = −0.14, *p* = 0.03).

It was further found that synchronicity MD was significantly and positively correlated with presence of MIL (*r* = 0.28, *p* < 0.001(and anxious attachment to God (*r* = 0.24, *p* < 0.001(. Synchronicity MD was significantly and negatively correlated with avoidant attachment to God (*r* = −0.21, *p* = 0.001). A sense of MIL was significantly and negatively correlated with avoidant attachment to God (*r* = −0.50, *p* < 0.001). No significant correlations were found between presence of MIL and anxious attachment to God. Finally, anxious attachment to God was negatively correlated with avoidant attachment to God (*r* = −0.31, *p* < 0.001; see Table 2).

The study's hypotheses were tested using 10,000 bias-corrected bootstrapped samples and 95% confidence intervals, following the recommendations of Aguinis et al. (2017), Hayes (2009), and Hayes (2012), multiple mediation analysis outline. Since our aim was to test the directional hypotheses among the constructs rather than to re-evaluate the psychometric properties of the scales, which had already been established in prior studies, we conducted path analysis with manifest variables, based on aggregated scale scores (e.g., Granqvist et al., 2012; Littman-Ovadia and Steger, 2010; Russo-Netzer and Icekson, 2023). Moreover, given the sample size and model complexity, path analysis with manifest variables provided a more parsimonious and statistically stable approach (Hayes et al., 2017; Williams et al., 2009). Avoidant attachment to God and anxious attachment to God were exogenous variables, while SA and synchronicity MD were the mediators, and MIL was the endogenous variable (see Figure 1). Testing of the suggested full model showed very good fit [χ^2^ (1) = 0.973, *p* = 0.324, *NFI* = 0.996, *TLI* = *1.001, CFI* = 1.000, *RMSEA* = 0.000, *SRMR* = 0.01, *ECVI* = 0.162], meaning that this model provided an excellent starting point for further analysis.

Although the fit of the hypothesis model was very good, examination of the separate paths revealed that avoidant attachment to God and anxious attachment to God were not significantly related to SA and avoidant attachment to God was not significantly related to synchronicity MD. Therefore, these paths were removed from the final model (see Table 4). Removing statistically non-significant paths while transparently reporting both the original and the adjusted model is an accepted and recommended practice in mediation and path analysis research (e.g., Rohrer et al., 2022; Schuler et al., 2025).

**Table 4:** Path analysis for the full research model.

	***b***	***se***	**β**	***p-value***	***LLCI***	***ULCI***
Attachment anxiety → Synchronicity awareness	0.087	0.055	−0.112	0.093	−0.03	0.24
Attachment avoidance → Synchronicity awareness	−0.058	0.035	0.106	0.113	−0.25	0.02
Synchronicity awareness → Synchronicity meaning-detection	0.784	0.062	0.614	< 0.001	0.52	0.70
Attachment anxiety → Synchronicity meaning-detection	0.134	0.053	0.128	0.011	0.03	0.22
Attachment avoidance → Synchronicity meaning-detection	−0.054	0.034	−0.081	0.112	−0.18	0.02
Synchronicity meaning-detection → Presence of meaning in life	0.254	0.063	0.222	< 0.001	0.11	0.33
Attachment anxiety → Presence of meaning in life	−0.277	0.068	−0.232	< 0.001	−0.34	−0.13
Attachment avoidance → Presence of meaning in life	−0.398	0.043	20.523	< 0.001	−0.62	−0.42

Explained variance in the model: R^2^ for synchronicity awareness was 0.03, R^2^ for synchronicity meaning-detection was 0.44, and R^2^ for presence of meaning in life was 0.33.

The fit of the final model was excellent, and all paths were statistically significant. Table 5 details all standardized, unstandardized, and significance levels of the model coefficients. The final model had fit indices that met criteria for excellent model fit: [χ^2^ (2) = 3.479, *p* = 0.176, *NFI* = 0.987, *CFI* = 0.994, *TLI* = 0.971, *RMSEA* = 0.055, *SRMR* = 0.02, *ECVI* = 0.164]. The trimmed model was significantly superior to the full model, since an increase in degrees of freedom was coupled with a non-significant chi-square change: Δχ^2^(1) = 2.506, *p* > 0.05.

**Table 5:** Path analysis for the final research model.

	***b***	***se***	**β**	***p-value***	***LLCI***	***ULCI***
Synchronicity awareness → Synchronicity meaning-detection	0.795	0.062	0.622	< 0.001	0.52	0.70
Attachment anxiety → Synchronicity meaning-detection	0.159	0.051	0.152	0.002	0.06	0.25
Synchronicity meaning-detection → Presence of meaning in life	0.254	0.062	0.224	< 0.001	0.11	0.33
Attachment anxiety → Presence of meaning in life	−0.277	0.068	−0.232	< 0.001	−0.34	−0.13
Attachment avoidance → Presence of meaning in life	−0.398	0.042	−0.528	< 0.001	−0.62	−0.42

Explained variance in the model: R^2^ for synchronicity meaning-detection was 0.44, and R^2^ for presence of meaning in life was 0.32.

**Figure 2:**
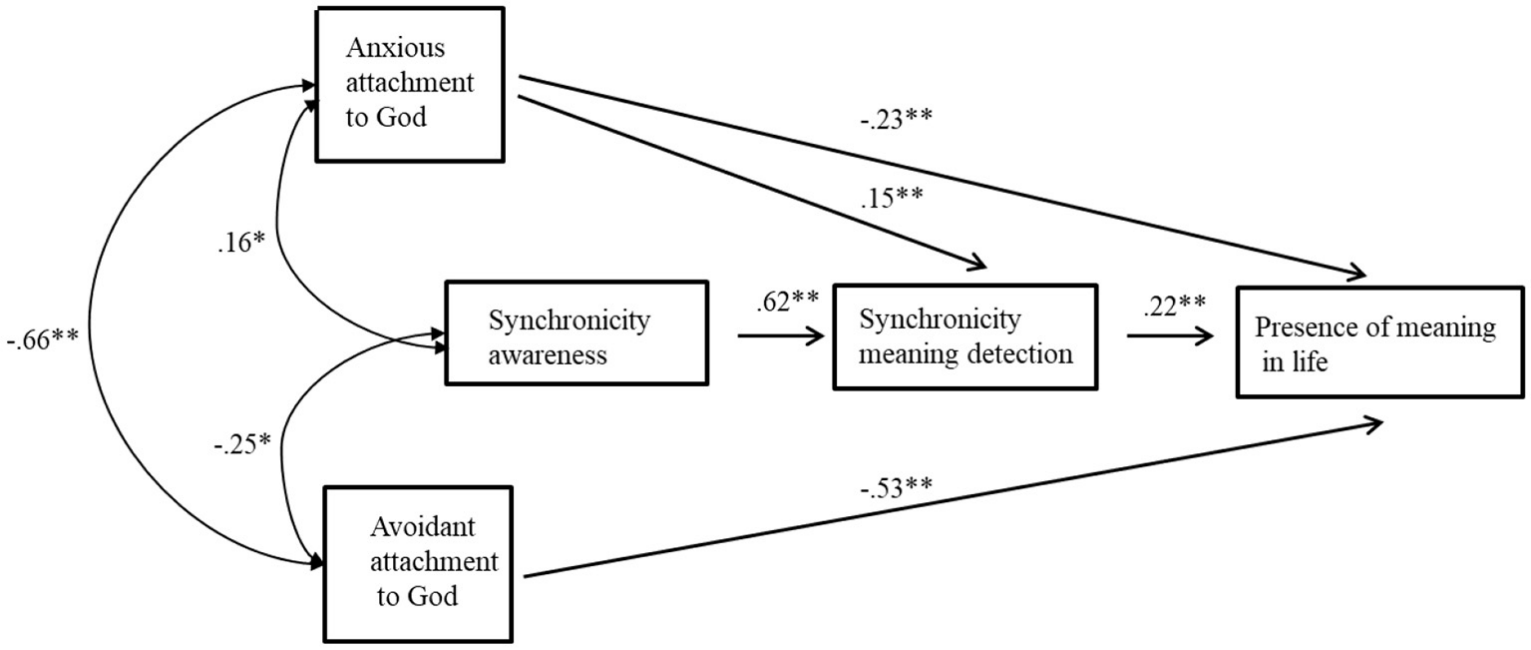
The final model. Numbers above lines indicate standardized path coefficients and significance values. * p < 0.05 **p < 0.001, *N* = 242.

The findings (displayed in Table 5) support both hypotheses: H1 that anxious attachment to God is directly and negatively associated with presence of MIL [β = −0.23, *SE* = 0.07, *p* = 0.00, 95%CI (−0.34, −0.13)] and H2 stating the same for avoidant attachment to God [β = −0.53, *SE* = 0.04, *p* = 0.00, 95%CI (−0.62, −0.42)]. Testing for the indirect effects between anxious attachment to God and MIL in the model via the bootstrapping procedure (Hayes, 2009) yielded two significant indirect effects. The indirect effect of SA to MIL through synchronicity MD was found to be significant: *b* = 0.20, β = 0.14, *SE* = 0.06, 95%CI [0.07, 0.22], thus partly supporting H3 and H4. The indirect effect of anxious attachment to God on MIL through synchronicity MD was also found to be significant: *b* = 0.04, β = 0.03, *SE* = 0.02, 95%CI [0.01, 0.07], supporting H5. H6 was not supported. See Figure 2 for the final model paths with their standardized coefficients.

## 4 Discussion

This study sought to explore cultural variations in the sense of MIL by comparing ultra-Orthodox religious individuals with their secular counterparts. By delving into these distinct groups, the research aimed to uncover how different worldviews shape the sense of meaning people derive from their lives. Additionally, the study addressed several underexplored factors that might contribute to these experiences, including the role of attachment to God, as well as individuals' awareness of synchronicity and their ability to detect meaning in everyday occurrences. Through this investigation, the study provides new insights into how these elements interact and influence the overall experience of meaning, shedding light on the complex interplay between cultural context and personal perception.

The findings suggest significant differences between ultra-Orthodox and secular individuals as to anxious and avoidant attachment styles. Ultra-Orthodox individuals were found to exhibit a more anxious attachment style to God, while secular individuals tended to display a more avoidant attachment style. These findings can be explained through a combination of theological, cultural, and psychological factors. In ultra-Orthodox communities, God is often perceived as an intimately involved and omnipotent presence, deeply integrated into every aspect of life (Silberman, 2005). This perception fosters a personal relationship with God that can mirror human attachment styles, with anxious attachment characterized by a strong desire for closeness, fear of abandonment, and concerns about one's worthiness in the eyes of God (e.g., Cherniak et al., 2021; Granqvist et al., 2012). Research indicates that individuals with anxious attachment to God often seek reassurance through prayer, rituals, and adherence to religious norms, reflecting a need to maintain and secure this divine relationship. This attachment style appears to be reinforced by a religious framework that emphasizes God's omnipresence and active involvement in one's daily life, leading to heightened dependency and emotional investment in this divine connection (Granqvist, 2020; Kirkpatrick, 2005).

Another possible explanation for the heightened levels of anxiety attachment style reported by ultra-Orthodox individuals is the high standard of religious observance among ultra-Orthodox communities, fostering an environment of stringent expectations. In such a community, the pressure to meet these standards can intensify fears of divine disapproval or punishment, contributing to an anxious attachment style, marked by a heightened need for validation and reassurance from God (e.g., Tung et al., 2018). This sense of anxiety is not only a personal concern but is often reinforced by the social structure of the community, where adherence to religious norms is closely monitored and deviations are discouraged (Klonover et al., 2023; Russo-Netzer and Bergman, 2020). In a broader sense, the communal emphasis on piety and moral behavior can amplify anxiety, as individuals continuously strive to meet both divine and communal expectations (Exline et al., 2011).

Conversely, secular individuals tend to exhibit a more avoidant attachment style to God, reflecting broader secular attitudes toward religion often characterized by distance, skepticism, or disinterest. Avoidant attachment to God involves emotional disengagement and a reluctance to depend on or to seek comfort from a divine figure. For many secular individuals, God may be seen as distant, uninvolved, or irrelevant, which aligns with a preference for autonomy and self-reliance over reliance on a higher power (Kirkpatrick and Shaver, 1992). This avoidant stance is often underpinned by secular values that prioritize independence and personal control, reducing the need for a close relationship with a deity (Granqvist and Hagekull, 2000).

Ultra-Orthodox and secular individuals reported similar levels of SA as well as MD, suggesting that regardless of religious background, this phenomenon is quite widespread. This further corroborates previous findings regarding the scope and prevalence of the phenomenon among non-clinical general population samples (e.g., Fach et al., 2013; Russo-Netzer and Icekson, 2023). Some studies, though, have suggested that SA may be positively related to the level of religiosity (e.g., Coleman et al., 2009). Rosenstreich et al. (2024), which included participants from the US and the UK, revealed a small positive association between both SA and MD and religiosity. One possible explanation for the inconsistent patterns of relationship between religiosity and SA may reflect a distinction between the frequency of such experiences and the subjective phenomenological interpretation granted by individuals. Thus, the encounter with synchronicity experiences may represent a universal, widespread human experience (Coleman et al., 2009), which is not necessarily bounded to a specific religious or cultural background. However, such background may shape the ways in which individuals make sense of these experiences as part of a larger cultural “web” of meanings in their lives (Russo-Netzer and Mayseless, 2021). (Russo-Netzer and Icekson 2020) conducted an in-depth qualitative exploration of synchronicity experiences, suggesting that sociocultural context may play a crucial role in shaping how both secular and religious individuals interpret synchronicity with participants with religious beliefs viewing synchronicity as affirmations of their faith and overarching worldview and attributing such moments to divine providence or personal guidance. Future studies should further expand the exploration of synchronicity experiences to include varied religious tendencies and sociocultural backgrounds.

The findings also suggest that ultra-Orthodox individuals experience more MIL than secular individuals. This finding may be explained through several interconnected factors related to purpose, community, structure, and values. Ultra-Orthodox individuals' lives are guided by a clear framework of religious teachings, rituals, and community expectations that provide direction and purpose (Russo-Netzer and Bergman, 2020). This is supported by research suggesting that having a coherent sense of purpose, particularly one grounded in spiritual or religious beliefs, is strongly associated with higher levels of MIL (Cohen and Rozin, 2001; Park, 2005).

Furthermore, the ultra-Orthodox lifestyle is deeply embedded within close-knit communities that offer strong social bonds and support systems (e.g., Russo-Netzer and Bergman, 2020). These communities provide a profound sense of belonging and interconnectedness, enhancing individuals' overall sense of purpose and meaning (Krause, 2015). Social support and community involvement may function as significant contributors to perceived MIL, as they fulfill basic psychological needs for connection and belonging (Ryan and Deci, 2000). Secular individuals often navigate more individualistic environments, which may lack the same depth of communal ties and support structures (Russo-Netzer, 2016; Zuckerman et al., 2016).

Another key factor is the highly structured nature of ultra-Orthodox life, which centers around daily routines of prayer, study, and religious observance. This structured framework imparts a sense of order and direction, engaging individuals in meaningful activities and habits (Heintzelman and King, 2014). For example, regular practices such as expressing gratitude can enhance wellbeing (Russo-Netzer et al., 2021). Moreover, these consistent religious rituals continually reinforce one's values and purpose, contributing to a stable sense of identity and meaning (Silberman, 2005).

Ultra-Orthodox beliefs also often involve a sense of transcendence, connecting individuals to something greater than themselves, such as God or a divine plan. This connection can provide a profound sense of existential meaning that secular individuals, who may lack such a framework, might struggle to find (Park, 2005). Religion offers answers to existential questions about life, death, and purpose, reducing existential anxiety and enhancing perceptions of meaning (Pargament, 2011; Pirutinsky, 2009). Finally, ultra-Orthodox communities place a strong emphasis on family values, tradition, and the passing down of beliefs from one generation to the next, which can foster a sense of legacy and purpose beyond oneself (e.g., Ganz, 2024).

The findings also point to a rather new direction in understanding the relationship between attachment to God and MIL. Insecure attachment to God (avoidant or anxious) was negatively associated with MIL. This finding provides further support for the religion-as-an-attachment process (Kirkpatrick and Shaver, 1992; Kirkpatrick, 2005; Shaver and Mikulincer, 2012), suggesting that an unstable relationship with the transcendent may lead to a decreased sense of MIL. Given that an essential component of MIL is a sense of coherence, connection, and order, an inconsistent, unsteady, and uncertain connection to the transcendent may fail to serve as a trustworthy source of meaning (George and Park, 2016; Heintzelman and King, 2014; Martela and Steger, 2016).

Further, the present findings emphasize the eudaimonic aspect of wellbeing, particularly the sense of MIL, through the lens of attachment to God (e.g., Culver and Lundquist Denton, 2017; Keefer and Brown, 2018; Stroope et al., 2013). This dimension of wellbeing has been relatively underexplored in previous research, which primarily focused on mental health outcomes, quality of life, and psychopathology (e.g., Bradshaw and Kent, 2018; Granqvist et al., 2012; Hiebler-Ragger et al., 2016; Stulp et al., 2019; Tung et al., 2018). These earlier studies tended to overlook the role that attachment to God and a sense of MIL can play in an individual's overall wellbeing.

The negative relationship between insecure attachment to God and sense of MIL may also be considered an expression of a spiritual struggle: “experiences of tension, conflict, or strain that center on whatever people view as sacred” (Pargament and Exline, 2022, p. 6). Spiritual struggles, doubting one's faith, or experiencing conflict with one's beliefs, are directly linked to reduced MIL because they challenge the core aspects of a person's spiritual worldview (Exline et al., 2014). These struggles may undermine the sense of coherence, purpose, and connection that typically arises from secure spiritual beliefs. Rather than providing a source of comfort, guidance, and meaning, an insecure attachment to God can become a source of internal conflict and existential distress.

Put differently, insecure attachment to God, whether anxious or avoidant, often involves feelings of distrust, fear, uncertainty, or emotional distance from God (Cherniak et al., 2021; Kirkpatrick, 2005). These attachment styles mirror the difficulties individuals face in their human relationships and project them onto their spiritual relationship with God, leading to a strained and conflict-ridden connection. This strained relationship can contribute to spiritual struggles, where individuals grapple with doubts, fears of divine abandonment, or feelings of unworthiness, all of which undermine a stable sense of MIL (Exline, 2013; Pargament and Exline, 2022).

While most previous studies validated the connection between interpersonal attachment styles and meaning (e.g., Shaver and Mikulincer, 2012; Dewitte et al., 2019), the current study broadens the scope of our understanding to include attachment to God as an independent source of MIL. An anxious attachment to God involves a persistent fear of divine rejection, disapproval, or punishment. People with this attachment style may constantly seek reassurance through religious practices but remain doubtful of God's love or acceptance. This emotional instability can erode the sense of meaning, as the individual is caught in a cycle of seeking and fearing God's presence, unable to experience the comfort and purpose that a secure attachment to God typically provides. Instead of feeling supported by a benevolent higher power, the anxious individual may feel perpetually judged and anxious, hindering their ability to derive a stable sense of meaning from their spirituality. Similarly, an avoidant attachment to God reflects a spiritual struggle characterized by emotional distancing, mistrust, and reluctance to engage with the divine. Avoidant individuals often view God as distant, uninvolved, or untrustworthy. The lack of a deep, meaningful connection with the divine can leave avoidant individuals feeling a diminished sense of meaning (Bryant and Astin, 2008). Future studies should validate these preliminary findings and further explore their potential interplay.

Findings of the mediation analyses suggest that synchronicity MD significantly mediated the association between anxious attachment to God and MIL. This suggests that individuals with anxious attachment to God tend to interpret synchronistic events as meaningful, which, in turn, enhances their overall sense of MIL. This finding aligns with previous research indicating that anxious attachment style may drive individuals to seek more personal or spiritual signs to mitigate their anxiety (e.g., Henderson and Kent, 2022; Granqvist et al., 2010). This finding is also consistent with the idea that religious doubts and questioning might be part of a healthy process and suggests that meaning-making of unordinary experiences regulates the impact of religious doubt on MIL, as suggested by studies on quest approaches to religion (Exline et al., 2014). The positive relationship between synchronicity MD and MIL also corroborates previous preliminary findings (Russo-Netzer and Icekson, 2023). In a broader sense, it can be suggested that meaning-making processes of unexpected coincidences may regulate the impact of doubtful connection with God on MIL: the higher the anxiety level, the more benefits of meaning it brings through synchronicity experiences. This direction should be further explored and validated in future studies.

Interestingly, SA was not found to significantly mediate the association between anxious attachment to God and synchronicity MD. It seems that it is not merely the recognition of synchronistic events but the interpretation of these events that plays a critical role in fostering a sense of MIL for individuals with anxious attachment styles. SA refers to an individual's general sensitivity to noticing coincidences or events that seem meaningful. However, noticing such events alone does not necessarily provide emotional or existential comfort. It is a more passive form of awareness that does not always result in a deeper sense of meaning. In contrast, synchronicity MD involves actively interpreting these events as meaningful or as signs that resonate with personal beliefs, particularly spiritual or religious frameworks (Rosenstreich et al., 2024; Russo-Netzer and Icekson, 2020). This active interpretation may provide a sense of purpose and direction that may be especially important for individuals with anxious attachment?

Another significant effect refers to the relationship between SA and MIL, mediated by synchronicity MD. Along the lines of the REM model (Russo-Netzer and Icekson, 2020), the process of detecting synchronicity goes beyond passive awareness. It involves active cognitive engagement, where individuals consciously connect their unexpected events with their personal beliefs or spiritual or cultural frameworks. This active engagement in the recognition of significant patterns allows for the construction of a meaningful narrative, offering individuals emotional support and validation (Jung, 1969; Roxburgh et al., 2018). This interpretation may lead to enhanced MIL, as individuals weave these experiences into their personal narratives (e.g., Russo-Netzer and Icekson, 2020). Overall, the interplay between SA and MD of such experiences appears to be an active, complex process, involving pattern recognition and personal/cultural interpretations, which calls for further unpacking in future studies.

Avoidant attachment to God was not significantly related to SA and MD. Thus, SA and MD did not mediate the relationship between avoidant attachment to God and MIL. Previous research suggests that individuals with interpersonal avoidant attachment styles tend to be less exploratory in their environment and relationships (Mikulincer and Shaver, 2007). The reduced tendency to explore could lead to fewer opportunities to notice and interpret synchronistic events. Further, studies have shown that individuals with avoidant attachment styles tend to score lower on measures of mindfulness (Pepping et al., 2014) and openness to experiences (Marrero-Quevedo et al., 2019). Given that SA typically requires a degree of present-moment awareness and openness to experience (e.g., Russo-Netzer and Icekson, 2023), higher avoidance style toward God may be less relevant to SA and MD tendencies.

### 4.1 Limitations and recommendations for future studies

The present study has limitations to consider. Data were collected only from self-report surveys, which are considered suitable for assessing subjective experiences but may lead to bias in participant responses. To overcome this, we used procedural design methods (confidentiality and anonymity, separate questionnaire sections and instructions, etc.; Podsakoff et al., 2003), but future research could use other sources (such as brain and behavioral measures; e.g., Rominger et al., 2023, 2024b). Also, the present findings are correlational, thus future causal directionality must be tested through longitudinal designs, intervention, or experimental research in order to further validate the results, as well as their implications. For example, future research could use daily diary methods (e.g., Rominger et al., 2024a). In addition, it should be noted that the matching procedure was limited to age and gender, and did not account for other potential confounding variables. Moreover, we did not test for measurement invariance of the attachment to God scales across groups. Thus, the validity of the group comparisons should be interpreted with caution, as the meaning of these constructs may vary between secular and ultra-Orthodox participants. Finally, the analyses were conducted with manifest variables rather than latent variables, thus the associations may be somewhat conservative and should be interpreted with this limitation in mind.

Despite these limitations, this study makes several contributions. It adds to the literature on MIL by expanding the consideration of sociocultural contexts in comparing secular and ultra-Orthodox individuals, illustrating how religious affiliation is associated with a sense of connection to the divine and offering a more comprehensive view of how meaning is constructed and experienced across different sociocultural settings. It also advances our understanding of attachment and religion-as-an-attachment theories by highlighting the nuanced role of attachment to God styles and MIL, deepening our grasp of how different attachment styles to God interact with aspects like SA and MD and emphasizing the significance of exploring attachment to God within the context of attachment theory. Finally, it extends existing literature of clinical reports and case studies on the phenomenon of SA to better understand the cultural and relational mechanisms underlying it.

## Data Availability

The raw data supporting the conclusions of this article will be made available by the authors, without undue reservation.
